# Estimation of the Heteroskedastic Canonical Contagion Model with Instrumental Variables

**DOI:** 10.1371/journal.pone.0168967

**Published:** 2016-12-28

**Authors:** André L. P. Ribeiro, Luiz K. Hotta

**Affiliations:** 1 Banco Itaú-Unibanco, São Paulo SP, Brazil; 2 Department of Statistics, University of Campinas, Campinas SP, Brazil; East China University of Science and Technology, CHINA

## Abstract

Knowledge of contagion among economies is a relevant issue in economics. The canonical model of contagion is an alternative in this case. Given the existence of endogenous variables in the model, instrumental variables can be used to decrease the bias of the OLS estimator. In the presence of heteroskedastic disturbances this paper proposes the use of conditional volatilities as instruments. Simulation is used to show that the homoscedastic and heteroskedastic estimators which use them as instruments have small bias. These estimators are preferable in comparison with the OLS estimator and their asymptotic distribution can be used to construct confidence intervals.

## Introduction

The spread of a crisis from one economy to other economies is called contagion. This issue has been addressed recently in several studies due to its great importance to decisions and resource allocations for risk management. Various definitions of contagion have been proposed but no definition has been universally accepted.

[1] proposed the canonical model of contagion, which does not require a priori knowledge of the periods of crisis. The model is able to identify them, and also gives a direct interpretation of contagion. However, the canonical model suffers from the endogeneity problem of the regressor variables, which leads to bias of the ordinary least squares (OLS) estimates of the parameters that indicate contagion. To solve this problem [1] proposed to use valid instrumental variables (IVs) from economic theory. In their application, they considered the conditional variance of the error term as constant. This is generally true when quarterly data are used, as in [1] and [2], but this generally does not hold in daily financial data. [3] applied the canonical model to daily data and also considered the GARCH model for the error term, but, he considered the same instruments as [1] and [2]. There are many works in the literature that use the heteroskedasticity of the error as instruments, but they consider the contemporaneous covariance matrix of the error terms. See, for instance, [4] and [5] for the general case, and [6] for the canonical model of contagion. [7] considered the canonical model of contagion in the presence of conditional heteroskedastic errors. They showed that the conditional variance is a valid instrumental variable and applied the estimation method to model the contagion among some Asian countries. However, they did not show the sample properties of the proposed instruments and the present paper tries to fill this gap. The instruments proposed here have some similarity with the approach suggested by [5]. There are also papers related to the use of heteroskedasticity in simultaneous equation system dealing with the related problem of identification (see, for instance, [8] and [9]).

[10] noted that in the presence of weak instruments, the asymptotic approximations are not valid even for large samples. [11] proposed a test, which was also studied by [12], to identify weak instruments with the the null hypothesis that the instruments are weak. Other tests to detect weak instruments have been proposed, such as [13]’s test, which was also studied by [14]. The properties of the limited information maximum likelihood estimator (LIML), in the context of many instruments, were studied by [15]. [16] investigated two-stage and LIML IV estimators, whereas we have only one endogenous variable. Another study on the same subject was carried out by [17]. The estimator considered in this work is two-stage when the variance-covariance matrix of errors is considered homoscedastic and heteroskedastic.

The main contributions of this paper are: a) to show that the estimated volatility of the error terms are valid instruments and, more importantly, that the estimators, which consider the volatilities to be instruments, have small bias; and b) to show that the confidence intervals based on the asymptotic estimator distribution have almost the right coverage. The paper is organized as follows. The next section presents the canonical model of contagion and the problem of endogeneity of the explanatory variables. Then, we present a brief review of the IV theory, with emphasis on estimation and tests to detect weak instruments. Following we propose to use the volatility as instruments and uses simulation to show that the proposed variables are valid instruments. Next, we present three possible estimation methods and uses simulations to study the properties of these estimators. It shows that the proposed estimators, based on the volatilities as instruments, are able to estimate the contagion parameter. Finally, we concludes.

## Canonical Model of Contagion

Standard economic theory relates the performance of a given economy to its macro and microeconomics foundations. Thus, an econometric model must take into account the macro and microeconomic structures. Denoting *y*_*it*_ as the variable that measures the performance of the i-th country, *i* = 1, …, *N*, in the period *t* (*t* = 1, …, *n*), the performance is explained by economic variables which are common to all economies, called observed common factors, denoted by the *s* × 1 vector *x*_*t*_, and economic variables specific to each economy, called observed specific factors, denoted by the *k*_*i*_ × 1 vectors *x*_*it*_, : *i* = 1, …, *N*. The performance indicator variables, *y*_*it*_, can be, for instance, stock market indexes, interest rates of public securities and exchange rates, among others that are used in literature. Examples of common variable are commodity prices, and of specific factors are inflation indexes and export levels. The canonical model of contagion, based on [1], and further examined by [2], is given by

$$\begin{array}{ccc} y_{it} & = & \delta_{i}^{T}x_{t}+\alpha_{i}^{T}x_{it}+\beta_{i}I\left(\sum_{j=1,j\neq i}^{N}I\left(-y_{jt}-c_{j}\right)\right)+u_{it}, \end{array}$$ (1)

where *δ*_*i*_ is the *s* × 1 vector of the unknown parameter that determines the influence of common factors in the country *i*, *α*_*i*_ is the *k*_*i*_ × 1 vector of unknown parameters that determines the influence of specific factors in country *i*, and *u*_*it*_ is the mean zero error term of country *i* in period *t*. Denoting *u*_.*t*_ = (*u*_1*t*_, …, *u*_*Nt*_)^*T*^, we have that *u*_.*t*_ is a random variable with zero conditional mean (*E*(*u*_.*t*_|Ω_*t*_) = 0) and conditional covariance matrix Ω_*t*_ ($Var\left(u_{.t}|F_{\text{t}-1}\right)=\Omega_{t}$), where $F_{\text{t}-1}$ is the information available up to time *t* − 1. Ω_*t*_ is an *N* × *N* matrix with element [*σ*_*ijt*_], which follows a multivariate volatility model and *c*_*j*_ *j* = 1, …, *N*, are threshold values for the crisis defined in an *ad hoc* way.

Contagion is the dependence in moments of crisis among countries that cannot be explained by macroeconomic fundamentals. Therefore, when economy *j* is in crisis, the other economies will change their states; for example, the equilibrium performance of countries *i* ≠ *j* will change their levels. We define an economy as being in crisis when *y*_*jt*_ is smaller than a certain lower boundary, (−*c*_*j*_). This will define an indicator variable, *I*(−*y*_*jt*_ − *c*_*j*_), which is equal to 1 when economy *j* is in crisis, and 0 otherwise. *I*(*x*) is the indicator function, which is equal to 1 if *x* > 0, and 0 otherwise.

The use of ordinary or weighted least squares techniques to estimate the model is not appropriate because as pointed out by [1], the estimators of *β*_*i*_ are inconsistent due to the endogeneity of the indicator variable, $I\left(\sum_{j=1,j\neq i}^{N}I(-y_{jt}-c_{j})\right),$ which is correlated with the error *u*_*it*_ by construction.

## Estimation with Instrumental Variables

### Introduction

Consider the general single-equation linear model, where for simplicity we have dropped the equation subscript *i*:

$$\begin{array}{ccc} y & = & X\alpha +Z\beta +u, \end{array}$$ (2)

where $y\in {\mathbb{R}}^{n}$; $X\in {\mathbb{R}}^{n\times k}$ and $Z\in {\mathbb{R}}^{n\times q}$ are the observed variables; $\alpha \in {\mathbb{R}}^{k}$ and $\beta \in {\mathbb{R}}^{q}$ are unknown parameters; and *u* is a vector of random unobservable errors with mean zero and with *E*(*uu*^*T*^) = Ω. *X* is the matrix of exogenous variables, i.e., uncorrelated with the error term *u*, while *Z* is the matrix of endogenous variables, i.e., correlated with the error term *u*. In the canonical model of contagion Eq (1), for each equation we have that *X* is given by the variables *x*_*t*_ and *x*_*it*_ and *Z* by the indicator function.

Denote *B* = [*X* : *Z*] as the regressor matrix of size *n* × (*k* + *q*) and *η* = vec([*α* : *β*]) as the vector of unknown parameters of size (*k* + *q*) × 1. The operator vec transforms a matrix *a* × *b* into a vector *ab* × 1 by piling the columns of the matrix. Then, Eq (2) can be written as

$$\begin{array}{ccc} y & = & B\eta +u. \end{array}$$ (3)

Thus, we can work either with Eq (2) or Eq (3). We consider there is no perfect collinearity among the regressor variables, i.e., rank of *B* is (*k* + *q*).

Consider the linear model given by Eq (3), Ω = *σ*^2^ *I*_*n*_ with *σ*^2^ < ∞ with the assumptions: [*A*1] $\frac{1}{n}(B^{T}B)\overset{P}{\to }A$ finite and positive definite; [*A*2] $\frac{1}{n}(B^{T}u)\overset{P}{\to }0$; and [*A*3] $n^{-1/2}B^{T}u\overset{D}{\to }N(0,\sigma^{2}A)$. Then, the OLS estimator, given by ${\hat{\eta }}^{ols}=(B^{T}B{)}^{-1}B^{T}y$, is asymptotically normal, i.e., $n^{1/2}({\hat{\eta }}^{ols}-\eta )\overset{D}{\to }N(0,\sigma^{2}A^{-1}).$ See Section 4.2.2 of [18].

If we substitute *σ*^2^ and *A* by consistent estimators, the asymptotic distribution is still valid. In general, we take ${\hat{\sigma }}^{2}=(n-k-q{)}^{-1}(y-B{\hat{\eta }}^{ols}{)}^{T}(y-B{\hat{\eta }}^{ols})$ and $\hat{A}=\frac{1}{n}B^{T}B$.

When assumption [*A*2] does not hold, we have $\frac{1}{n}B^{T}u\overset{P}{\to }Q$ with *Q* ≠ 0. Thus, $plim_{n\to \infty }{\hat{\eta }}^{ols}=\eta +A^{-1}Q$, where the term *A*^−1^*Q* is responsible for the inconsistency of ${\hat{\eta }}^{ols}$. This problem can be tackled by IV methods.

### Estimators based on instruments

Assume there is a matrix $W\in {\mathbb{R}}^{n\times p}$, *p* ≥ *q*, where its columns are: (a) asymptotically not correlated with the error term *u*, and (b) correlated with *Z*, i.e., with the columns of *Z*. Then, the columns of *W* are instruments. Because the columns of the matrix *X* are uncorrelated with the error term, they are instruments. Therefore, Π = [*X*: *W*] is an *n* × (*k*+*p*) matrix of instruments. However, to avoid confusion we only refer to the variables that are columns of the matrix *W* as instruments, unless explicitly stated otherwise.

#### Homoscedastic IV estimator

The homoscedastic estimator of *η* is obtained by minimizing (*y* − *Bη*)^*T*^ *P*_Π_(*y* − *Bη*), where *P*_Π_ = Π(Π^*T*^ Π)^−1^ Π^*T*^. *P*_Π_ is the orthogonal projection matrix to the plane generated by the columns of Π. This brings us to the following estimator:

$$\begin{array}{ccc} {\hat{\eta }}^{giv} & = & {\left(B^{T}P_{\Pi }B\right)}^{-1}B^{T}P_{\Pi }y. \end{array}$$ (4)

See [18] (Eq 5.22) or [19] (Eq 8.29).

Consider the linear models given by Eq (3) with Ω = *σ*^2^*I*_*n*_ with *σ*^2^ < ∞ and consider the assumptions: [*B*1]: $\frac{1}{n}(\Pi^{T}\Pi )\overset{P}{\to }C$ finite and positive defined; [*B*2]: $\frac{1}{n}(B^{T}\Pi )\overset{P}{\to }D$ matrix of rank (*k* + *q*); [*B*3] $\frac{1}{n}(\Pi^{T}u)\overset{P}{\to }0$; and [*B*4] $n^{-1/2}(\Pi^{T}u)\overset{D}{\to }N(0,\sigma^{2})$. Then ${\hat{\eta }}^{giv}$ is asymptotically normal, i.e.,

$$\begin{array}{c} n^{1/2}\left({\hat{\eta }}^{giv}-\eta \right)\overset{D}{⟶}N\left(0,\sigma^{2}{\left(DC^{-1}D^{T}\right)}^{-1}\right). \end{array}$$

When we do not know *σ*^2^, *D*, and *C*, we estimate $Var({\hat{\eta }}^{giv})$ by ${\hat{\sigma }}^{2}(B^{T}P_{\Pi }B{)}^{-1}$, which is a consistent estimator if we use a consistent estimator for *σ*^2^. One consistent estimator is ${\hat{\sigma }}^{2}=\frac{1}{n}(y-B{\hat{\eta }}^{giv}{)}^{T}(y-B{\hat{\eta }}^{giv})$. See [18] (Theorem 5.2) or [19] (pp. 322–323). The estimate ${\hat{\eta }}^{giv}$ can be evaluated by the two-stage method (2SM) ([19], pp. 323–324).

Consider now the heteroskedastic linear model, i.e., the linear model given by Eq (3), with error term with known variance-covariance matrix Ω. Then under assumptions [*B*1] − [*B*3] and substituting [*B*4] by [*B*4*] $n^{-1/2}\Pi^{T}u\overset{D}{\to }N(0,E)$, where $\frac{1}{n}\Pi^{T}\Omega \Pi \overset{P}{\to }E$, ${\hat{\eta }}^{giv}$ is asymptotically normal, i.e.,

$$\begin{array}{c} n^{1/2}\left({\hat{\eta }}^{giv}-\eta \right)\overset{D}{⟶}N\left(0,{\left(DC^{-1}D^{T}\right)}^{-1}DC^{-1}EC^{-1}D^{T}{\left(DC^{-1}D^{T}\right)}^{-1}\right). \end{array}$$

The estimate of the variance estimator is given by

$$\begin{array}{c} \hat{Var}\left({\hat{\eta }}^{giv}\right)={\left(B^{T}P_{\Pi }B\right)}^{-1}B^{T}P_{\Pi }\hat{\Omega }P_{\Pi }B{\left(B^{T}P_{\Pi }B\right)}^{-1}, \end{array}$$ (5)

which is called robust estimation of the estimator variance. See [18] (Section 5.2.5) or [19] (p. 335).

#### Heteroskedastic IV estimator

The heteroskedastic IV estimator is the three-stage least squares estimator presented in Section 8.3.4 [18]. When the correlation matrix Ω is known, the heteroskedasticity IV estimator is obtained by minimizing (*y* − *Bη*)^*T*^ Π(ΠΩΠ)^−1^ Π^*T*^(*y* − *Bη*), which gives

$$\begin{array}{c} {\hat{\eta }}^{giv2}={\left(B^{T}\Pi {\left(\Pi^{T}\Omega \Pi \right)}^{-1}\Pi^{T}B\right)}^{-1}B^{T}\Pi {\left(\Pi^{T}\Omega \Pi \right)}^{-1}\Pi^{T}y. \end{array}$$

When the correlation matrix Ω is unknown, it is substituted by a consistent estimator. In our problem, considering that the error terms have small contemporaneous correlation, we estimate the matrix Ω by a diagonal matrix, with elements of the diagonal estimated by the square of the residuals of the 2SLS estimates, i.e., by ${\hat{u}}_{i}^{2}=\frac{1}{n}(y_{i}-B_{i.}{\hat{\eta }}^{vig}{)}^{T}(y_{i}-B_{i.}{\hat{\eta }}^{vig})$, where *B*_*i*._ is the i-th line of *B*.

Consider the linear model given by Eq (3), with variance-covariance matrix Ω ≠ *σ*^2^*I*_*n*_ known and consider the assumptions: [*C*1] $\frac{1}{n}\Pi^{T}\Omega \Pi \overset{P}{\to }E$ is finite and positively defined matrix; [*C*2] $\frac{1}{n}B^{T}\Pi \overset{P}{\to }D$ has rank (*k* + *q*); [*C*3] $\frac{1}{n}\Pi^{T}u\overset{P}{\to }0$; and [*C*4] $n^{-1/2}\Pi^{T}u\overset{D}{\to }N(0,E)$. Then ${\hat{\eta }}^{giv2}$ is asymptotically normal, i.e.,

$$\begin{array}{c} n^{1/2}({\hat{\eta }}^{giv2}-\eta )\overset{D}{\to }N(0,{\left(DE^{-1}D^{T}\right)}^{-1}). \end{array}$$

The variance of the estimator ${\hat{\eta }}^{giv2}$ is estimated by $(B^{T}\Pi (\Pi^{T}\hat{\Omega }\Pi {)}^{-1}\Pi^{T}B{)}^{-1}$.

In the heteroskedastic model, ${\hat{\eta }}^{giv}$ and ${\hat{\eta }}^{giv2}$ are both consistent and asymptotically normal. However, ${\hat{\eta }}^{giv2}$ is more efficient than ${\hat{\eta }}^{giv}$, in the sense that $Var({\hat{\eta }}^{giv})-Var({\hat{\eta }}^{giv2})$ is a positive semi-definite matrix.

### Test of weak instrumental variables

[12] developed a test statistic for the null hypothesis that the instruments are weak, based on the Cragg and Donald [11] statistic. The test of Stock and Yogo [12] classifies whether an instrument is weak according to the relative bias between the OLS estimator and the estimators based on the instruments. In this test, the null hypothesis that the instruments are weak is equivalent to saying that the maximum relative bias is larger than a fixed arbitrary value *r*. [12] presented a table of critical values for some values of *q*, *p*, *r*, and level of significance. When *q* = 1, the null hypothesis can be written as o $H_{0}:\frac{\left|E({\hat{\beta }}^{giv}-\beta )\right|}{\left|E({\hat{\beta }}^{ols}-\beta )\right|}\geq r$. In this table, for example, when *q* = 1, *p* = 3, *r* = 0.10 and at 5% level of significance we reject the null hypothesis when the Cragg-Donald statistic is larger than 13.91.

## Proposed Instrumental Variables

In the analysis of contagion, the variable generally used to measure the performance during a period is the return, during the same period, of a country’s stock market indicator; for example, the return of the S&P 500 index for the USA, or the Nikkei for Japan and the FTSE for the UK. A stylized fact of these return series, the *y*_*it*_ in the model, is that their conditional volatility changes over time. Hereafter, we call the conditional volatility just volatility. In the model, we have that the indicator variable, *I*(−*y*_*it*_ − *c*_*i*_), which indicates crisis, will be equal to one with larger probability in a period of high conditional volatility than in a period of low volatility. Thus, *σ*_*jt*_, the volatility for countries *j* = 1, …, *N*, *j* ≠ *i* can be used as instruments in the estimation and testing of the contagion parameter *β*_*i*_.

The following proposition shows that the volatility of *u*_*it*_ is an instrument for the canonical model of contagion.

**Proposition 1**: *In the canonical model of contagion given by* Eq (1), *for any i and j*, *we have*:

(a) $I\left(\sum_{j=1,j\neq i}^{N}I(-y_{jt}-c_{j})\right)$ *is correlated with u_it_*;
(b) *σ_jt_ is not correlated with u_it_*;
(c) *σ_it_ is correlated with* $I\left(\sum_{j=1,j\neq i}^{N}I(-y_{jt}-c_{j})\right)$.

The proof that *I*(−*y*_*jt*_ − *c*_*j*_) is correlated with *u*_*it*_ and *σ*_*it*_ can be found in [7] for *N* = 2 and time varying threshold values given by *c*_*j*_ *σ*_*jt*_, where *σ*_*jt*_ is the (conditional) volatility of *u*_*jt*_, but the proposition is also valid for *N* > 2 constant threshold values. The proposition states that the indicator function is an endogenous variable, and that the conditional volatility of country *i* (*σ*_*it*_) is an instruments and can be used to estimate *β*_*i*_. Similarly, we can prove that *ρ*_*ijt*_, the correlation between the errors is also a valid instruments.

### Simulation of Proposition 1

The magnitude of the correlations between the error terms and the endogenous variables indicates the influence on the bias, while the correlations between the instruments and the endogenous variables indicates the strength of the instruments. However, it is not easy to find analytical expressions for items (a) and (c) in Proposition 1 to evaluate the correlations. Fortunately, the correlations can be easily estimated by a Monte Carlo simulation. By simulating a large sample size we can estimate $Corr(I\left(\sum_{j=1,j\neq i}^{N}I(-y_{jt}-c_{j})\right),u_{it})$ and $Corr(I\left(\sum_{j=1,j\neq i}^{N}I(-y_{jt}-c_{j})\right),\sigma_{it})$ for *i*, *j* = 1, …, *N* almost without error.

In the simulation presented in this paper, the error follows a DCC-GARCH (1, 1) model for the regression error and threshold values *c*_*j*_ = 1.64, *j* = 1, …, *N*. Thus, the simulated data generating model used in this paper is:

$$\begin{array}{ccc} y_{it} & = & \delta_{i}+\alpha_{i}y_{i,t-1}+\beta_{i}I\left(\sum_{j=1,j\neq i}^{N}I\left(-y_{jt}-c_{j}\right)\right)+u_{it}, \end{array}$$ (6)

$$\begin{array}{ccc} u_{.t} & = & H_{t}^{1/2}\epsilon_{t};\;\epsilon_{t}∼iid\left(0,I_{N}\right) \end{array}$$ (7)

$$\begin{array}{ccc} H_{t} & = & D_{t}P_{t}D_{t};\;D_{t}=diag\left(\sigma_{it}\right) \end{array}$$ (8)

$$\begin{array}{ccc} \sigma_{it}^{2} & = & a_{i0}+a_{i1}u_{i,t-1}^{2}+a_{i2}\sigma_{i,t-1}^{2} \end{array}$$ (9)

$$\begin{array}{ccc} P_{t} & = & {\left(Q_{t}⊙I_{N}\right)}^{-1/2}Q_{t}{\left(Q_{t}⊙I_{N}\right)}^{-1/2} \end{array}$$ (10)

$$\begin{array}{ccc} Q_{t} & = & \left(1-\theta_{1}-\theta_{2}\right)Q-\theta_{1}\epsilon_{t-1}\epsilon_{t-1}^{T}+\theta_{2}Q_{t-1}, \end{array}$$ (11)

where *Q* is the sample covariance of *ϵ*_*t*_; *a*_*i*0_, *a*_*i*1_, *a*_*i*2_, *θ*_1_, *θ*_2_ are all positive such that *a*_*i*1_ + *a*_*i*2_ < 1 ∀*i* = 1…, *N* and *θ*_1_ + *θ*_2_ < 1; and ⊙ is the Hadamard product. The Hadamard product is the product element by element of two matrices of the same order.

It is not easy to find analytical expressions for items (a) and (c) in Proposition 1 to evaluate the correlations. Fortunately, this can also be easily done by a Monte Carlo simulation. By simulating a large sample size, we can estimate $Corr(I\left(\sum_{j=1,j\neq i}^{N}I(-y_{jt}-c_{j})\right),u_{it})$ and $Corr(I\left(\sum_{j=1,j\neq i}^{N}I(-y_{jt}-c_{j})\right),\sigma_{it})$ for *i*, *j* = 1, …, *N* almost without error.

Four scenarios were used in the simulation, with *N* = 3. The values are given in Table 1.

**Table 1:** Parameters used in the simulation of Proposition 1. Four scenarios of the data generating model given by Eqs (6) to (11) with *N* = 3.

Scenario	i=	*δ*_*i*_	*α*_*i*_	*a*_*i*0_	*a*_*i*1_	*a*_*i*2_	*θ*_1_	*θ*_2_
1	1	8 × 10^−4^	0.02	10^−5^	0.107	0.864	0.03	0.96
	2	7 × 10^−4^	0.06	6 × 10^−6^	0.119	0.861	0.03	0.96
	3	3 × 10^−4^	-0.01	10^−6^	0.099	0.898	0.03	0.96
2	1	8 × 10^−4^	0.02	10^−5^	0.200	0.600	0.03	0.96
	2	7 × 10^−4^	0.06	6 × 10^−6^	0.100	0.800	0.03	0.96
	3	3 × 10^−4^	-0.01	10^−6^	0.099	0.898	0.03	0.96
3	1	8 × 10^−4^	0.02	10^−5^	0.106	0.864	0.20	0.70
	2	7 × 10^−4^	0.06	6 × 10^−6^	0.119	0.861	0.20	0.70
	3	3 × 10^−4^	-0.01	10^−6^	0.099	0.898	0.20	0.70
4	1	8 × 10^−4^	0.02	10^−5^	0.200	0.600	0.20	0.70
	2	7 × 10^−4^	0.06	6 × 10^−6^	0.100	0.800	0.20	0.70
	3	3 × 10^−4^	-0.01	10^−6^	0.099	0.898	0.20	0.70

In all of the scenarios given in Table 1 the elements of the matrix *Q* are: *q*_*ii*_ = 1, *q*_12_ = 0.5376, *q*_13_ = 0.5467 and *q*_23_ = 0.5922, where *q*_*ij*_ = *q*_*ji*_, *i*, *j* = 1, 2, 3. The scenarios differ in the temporal variability of the countries’ volatilities and temporal variability of the correlation between the error series (*u*_*it*_), because we want to study the effects of these factors on the quality of the instruments postulated. The basic scenario of this analysis is scenario 1, which replicates a model fitted by [20] to actual data rates of returns of the stock markets in Brazil, Mexico and the USA.

We simulated 50000 observations for each scenario considering there is no contagion, i.e., *β*_*i*_ = 0, *i* = 1, 2, 3. The percentage of crises for different countries and scenarios did not change much, varying from 3.70% to 4.43%. A country was contaminated by the others approximately 7.1% of the time and approximately 1.2% of the time at least two countries were simultaneously in crises. The volatility is higher in scenarios 1 and 3, where we gave less weight to the errors (*a*_*i*1_), and especially more weight to the past (*a*_*i*2_). In scenarios 3 and 4, the variation of the conditional correlation is larger than in scenarios 1 and 2, which is due to the parameters *θ*_1_ and *θ*_2_. When we gave more weight to the error term (*θ*_1_) and less to the past (*θ*_2_), we had a larger variability.

The twelve estimated correlations between the crisis indicator variables and the errors for the four scenarios are in the interval (−0.2996, −0.2678). The results strongly indicate that the indicator variable is endogenous, that is, the variables that indicate the existence of crisis in a block of economies is correlated with *u*_*jt*_. In all cases the p-values of the test of the null hypothesis that the correlation coefficient is zero (*H*_0_: *ρ* = 0) against the alternative hypothesis *H*_1_: *ρ* ≠ 0, is smaller than 10^−4^. The test of the hypothesis that *ρ* = 0 is based on Student’s test because we have a large sample and the dependence for two different times is small.

The estimates of the correlation between the postulated instruments and the endogenous variable (crisis indicator function) are around 0.10 or 0.20 depending on the scenario and the economy. The values are not negligible and the p-values to test the null hypothesis that the correlation is equal zero are always smaller than 10^−4^. In this regard, we expect that the proposed instruments are able to correct the bias of the OLS estimator. We also ran similar tests for other scenarios and different values of *c*_*i*_, such as *c*_*i*_ = 1.96 and *c*_*i*_ = 1.38, but the results were almost the same.

## Monte Carlo Study of the Estimators

### Introduction

In this section, we use a Monte Carlo simulation to compare the three estimators proposed for the contagion parameter *β*_*i*_ in the canonical contagion model: the OLS estimator, and the two estimators based on the IVs. The OLS estimation results are presented to show that the bias can be very large. We also test whether the confidence intervals based on the asymptotic distributions of the three estimators have the right coverage. The data generator process is that given by Eqs (6) to (11) with *N* = 4.

The proposed instruments are not observable and must be estimated. They are not exactly generated instruments, as defined in Section 6.1 of [18], because they must be estimated by the same model. Because of this, in Subsection Proposed estimators we propose an iterative method to find the IV estimates, and in Subsection Effect of the instruments’ estimation we show that the IV estimates using the exact volatility values and those using the estimation procedure are almost the same. The remainder of the section presents the comparison of the estimators.

We used the parameters found by [20] when fitting the DCC-GARCH(1, 1) model for a data set of stock market indexes from Argentina, Brazil, Mexico and the United States. The elements of the matrix *Q* are: *q*_*ii*_ = 1, *q*_12_ = 0.4948, *q*_13_ = 0.5382, *q*_14_ = 0.5472, *q*_23_ = 0.4352, *q*_24_ = 0.4275, *q*_34_ = 0.5941, where *q*_*ij*_ = *q*_*ji*_, *i*, *j* = 1, …, 4. The other parameters in the data generating process are given in Table 2.

**Table 2:** Parameters used in the simulation section. Data generating model given by Eqs (6) to (11) with *N* = 4.

Economy	*δ*_*i*_	*α*_*i*_	*α*_*i*0_	*α*_*i*1_	*α*_*i*2_	*θ*_1_	*θ*_2_
1	8 × 10^−4^	0.02	10^−5^	0.10	0.86	0.02	0.96
2	4 × 10^−4^	0.06	10^−5^	0.12	0.84	0.02	0.96
3	7 × 10^−4^	0.06	6 × 10^−6^	0.11	0.86	0.02	0.96
4	3 × 10^−4^	-0.01	10^−6^	0.10	0.88	0.02	0.96

### Proposed estimators

The homoscedastic IV estimate, ${\hat{\eta }}^{giv}$, is found iteratively in three steps as follows:

- (Step 1) Estimate models given by Eq (6) with *β*_*i*_ = 0, *i* = 1, …, *N* by OLS method and calculate the residuals ${\hat{u}}^{ols}$, where ${\hat{u}}_{it}^{ols}=y_{it}-{\hat{\delta }}^{ols}-{\hat{\alpha }}^{ols}y_{it},$ *i* = 1, …, *N*, *t* = 1, …, *n*;
- (Step 2) Fit a DCC-GARCH(1, 1) model (Eqs (7) to (11)) to ${\hat{u}}^{ols}$ and estimate the volatilities (${\hat{\sigma }}_{it}$);
- (Step 3) Estimate model (Eq (6)) *i* = 1, …, *N* by the IV method, with the instruments estimated in step (2).

If the estimates in steps (1) and (3) are close, we consider there is convergence. Otherwise we iterate steps (2) and (3) recursively until the estimates in two consecutive iterations of step (3) are close. At the end we have the homoscedastic IV estimate ${\hat{\eta }}^{giv}$. The iteration is necessary because the estimator in step (1) is biased, so it is expected that the residuals are also biased estimates of the error terms.

The heteroskedastic IV estimate is estimated as given in Subsection Heteroskedastic IV estimator, where the residuals are evaluated using ${\hat{\eta }}^{giv},$ found in the iterative procedure just described.

### Effect of the instruments’ estimation

**Fig 1:**
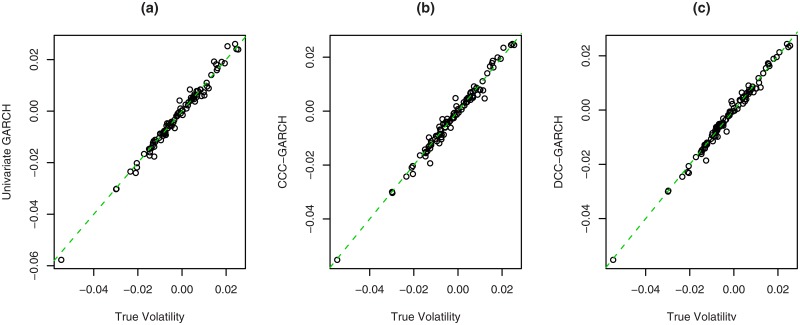
Scatterplot of the estimates of *β*_1_ by the heteroskedastic IV method, when the instruments are the true volatility against the estimates of *β*_1_ by the heteroskedastic IV method, when the instruments are the volatility estimated by: (a) univariate GARCH(1, 1) model, (b) multivariate CCC-GARCH, and (c) multivariate DCC-GARCH.

In this subsection we test whether the iteration of steps (2) and (3) overcomes the problem of bias, i.e., whether the estimated volatilities work as valid instruments. We showed in Section Proposed Instrumental Variables that the volatilities are valid instruments. Taking *β*_*i*_ = 0, *i* = 1, …, 4, we generated 100 replications of size 1000 using as data generating process the model given by Eqs (6)–(11). Then we estimated the model using the true volatility as instruments and the iterative three-step procedure. Fig 1 presents the scatterplot of the estimates of *β*_1_ using the true volatilities as instruments against the estimates when the instruments are estimated by three models: univariate GARCH(1, 1), CCC-GARCH(1, 1) and DCC-GARCH(1, 1). The difference between the estimates using the true volatility or any of the three estimates are negligible, especially compared to the variability of the estimates. Thus, there is strong evidence that the iterative procedure overcomes the problem of estimated instruments.

The result just presented can be very helpful in the study of the distribution of homoscedastic and heteroskedastic IV estimators of the contagion parameter. Because there is almost no difference between the estimates, we can use the true volatilities as instruments and we have the traditional estimates presented in Sections Homoscedastic IV estimator and Heteroskedastic IV estimator for the homoscedastic and heteroskedastic IV estimators, respectively.

### Properties of the OLS estimator and of the IV estimators

In this subsection we present the results of the Cragg-Donald test and the estimates of the bias and the mean square error (MSE) of the OLS estimator and the proposed heteroskedastic and homoscedastic IV estimators. The simulations are based on 1000 replications for different sample sizes and for the *i*-the equation we used the true volatilities *σ*_*jt*_, *j* ≠ *i*, *j* = 1, …, 4 as instruments. We have 1000 estimates of the parameter of interest *β*_*i*_, *i* = 1, …, 4, for each one of the three methods.

The results for the estimators of *δ*_*i*_ and *α*_*i*_, *i* = 1, …, 4, which are unbiased, because these parameters are not related to the endogenous variables of the model are not presented.

The results for the bias and the MSE for the three estimators are presented in Subsection Bias and mean square error of the estimators. Subsection Asymptotic confidence intervals analyzes the use of the asymptotic distribution to construct confidence intervals.

#### Bias and mean square error of the estimators

We first analyze how often the Gragg-Donald test rejects the null hypothesis that the relative bias is larger than *r*, with *r* equal to 0.05, 0.10 and 0.20 at 5% level of significance. The results are based on 1000 replications and the critical values are taken from Table 1 of [12] paper. Because the results are almost the same for the four economies, the discussion is in relation to the average rejection of the null hypothesis for the four economies. For sample size 500, the percentages of rejection of the null hypothesis are equal to (9.6, 26.5, 44.8) for *r* equal to 5, 10 and 20%, respectively. For sample size 1000, the percentages increased to (47.8, 75.9, 89.1), while for sample sizes 1500 and 2000, the percentages increased to (79.4, 94.6, 98.2) and (95.0, 99.1, 99.9), respectively. We can say that for sample size 500 we do not expect the bias to be small, but for sample size 1000 we already expect a substantial decrease in the bias, while for sample size 2000 the bias with the instrumental variables is almost always smaller than 5% of the bias of the OLS estimator.

Because the bias decreases substantially for sample sizes equal to 1500, we estimate the bias and MSE only for sample sizes equal to 100, 500 and 1000. Table 3 presents the estimated values of the bias and MSE of the three estimators of *β*_*i*_ considered in this paper, for sample sizes *n* equal to 100, 500 and 1000. We took *β*_*i*_ = 0, *i* = 1, …, 4.

**Table 3:** Estimates of the bias and MSE of the OLS estimator and the homoscedastic IV estimator (IV homo) and the heteroskedastic IV estimator (IV hete) of *β*_*i*_. In brackets the standard error of the estimate of the expected value of the bias and MSE. Results based on 1000 replications of the data generator model given by Eqs (6) to (11) and parameters from Table 2 and sample size *n*.

*n*			Economy 1	Economy 2	Economy 3	Economy 4
100	Bias	OLS	-0.0240	-0.0213	-0.0184	-0.0090
			(0.0004)	(0.0004)	(0.0003)	(0.0002)
		IV homo	**-0.0167**	**-0.0170**	**-0.0152**	**-0.0076**
			(0.0018)	(0.0020)	(0.0014)	(0.0007)
		IV hete	-0.0168	-0.0170	-0.0162	-0.0081
			(0.0018)	(0.0020)	(0.0014)	(0.0007)
	MSE	OLS	0.0007	**0.0006**	**0.0004**	**0.0001**
		IV homo	**0.0036**	0.0043	0.0022	0.0006
		IV hete	0.004	0.0044	0.0023	0.0006
500	Bias	OLS	-0.0239	-0.0211	-0.0186	-0.0090
			(0.0002)	(0.0002)	(0.0002)	(0.0001)
		IV homo	**-0.0025**	**-0.0028**	**-0.0034**	**-0.0013**
			(0.0006)	(0.0007)	(0.0006)	(0.0003)
		IV hete	-0.0029	-0.0033	-0.0035	-0.0014
			(0.0006)	(0.0007)	(0.0006)	(0.0003)
	MSE	OLS	0.0006	**0.0005**	0.0003	0.00009
		IV homo	0.0004	0.0005	0.0004	0.00008
		IV hete	**0.0004**	0.0005	**0.0003**	**0.00007**
1000	Bias	OLS	-0.0243	-0.0216	-0.0188	-0.0094
			(0.0002)	(0.0002)	(0.0001)	(0.0001)
		IV homo	**-0.0010**	-0.0009	**-0.0009**	**-0.0003**
			(0.0004)	(0.0005)	(0.0004)	(0.0002)
		IV hete	-0.0012	**-0.0007**	-0.0010	-0.0005
			(0.0004)	(0.0005)	(0.0003)	(0.0002)
	MSE		0.0004	0.0005	0.0003	0.00009
		IV homo	0.0002	0.0003	0.0001	0.00004
		IV hete	**0.0001**	**0.0002**	**0.0001**	**0.00003**

According to Table 3, the bias of the OLS estimators is significant, because in the absence of contagion, the estimated models indicate that the economy has an average decline of 2% in the performance index when one of the other economies is in crisis. This is in agreement with the previous theoretical results, which have suggested the use of instruments, as proposed by [1].

Table 3 presents the estimated bias for the homoscedastic and heteroskedastic estimators presented in Subsections Homoscedastic IV estimator and Heteroskedastic IV estimator. The analysis of the results shows that the bias of the IV estimators is smaller than the bias of the OLS estimator, especially for samples of moderate size, i.e., *n* = 500 and *n* = 1000. For the heteroskedastic IV estimator, the results are very similar to those found for the homoscedastic IV estimator. The bias, for moderately large samples, is almost nonexistent in comparison with the OLS estimator’s bias. Notice that the MSE of the heteroskedastic IV estimators for moderately large samples is smaller than the MSE of the homoscedastic IV estimators. These facts were already expected, as explained in Section Estimation with Instrumental Variables.

We ran a similar simulation with *β*_1_ ∈ {−0.05, −0.03, −0.01, 0, 0.01, 0.03, 0.05} and *β*_*i*_ = 0, *i* = 2, …, 4, with sample size 1000. We found similar results based in 1000 replications.

#### Asymptotic confidence intervals

The variances of the IV estimators for moderately large samples (*n* = 1000 and *n* = 500) are small, such that the estimators are unbiased or nearly unbiased, and with little probability of having a large error. An important point in inference is the construction of the confidence interval. In this subsection we test whether we can use the asymptotic Student-t distribution to construct confidence intervals in the usual way. We compare the coverage and the width of the intervals based on the homoscedastic and heteroskedastic IV estimators. The confidence interval based on the homoscedastic IV estimator is constructed using the robust estimator of the variance given by Eq (5).

We use the same data generating process given by Eqs (6) to (11), with the same parameter values. Using different values of *β*_1_ we find the confidence interval and test whether it covers the true value. We ran 1000 replications for each value of *β*_1_ ∈ {−0.1, −0.05, −0.03, −0.02, −0.01, 0, 0.01, 0.02, 0.03, 0.05, 0.1}, with sample size *n* = 1000. The estimated coverages of the 90% and 95% asymptotic confidence intervals are presented in Table 4. The estimated coverage using both estimators is almost equal to the nominal values, so we can say that in terms of coverage they are equivalent.

**Table 4:** Percentage of coverage of the (a) 90% and (b) 95% confidence interval. Results based in 1000 replications for each value of *β*_1_ in the model given by Eqs (6) to (7) with sample size equal to 1000.

		True values of *β*_1_										
		-0.10	-0.05	-0.03	-0.02	-0.01	0	0.01	0.02	0.03	0.05	0.10
90%	*homo*	0.91	0.91	0.90	0.90	0.90	0.91	0.90	0.90	0.90	0.90	0.89
	*hete*	0.90	0.90	0.90	0.90	0.90	0.90	0.90	0.89	0.90	0.90	0.90
95%	*homo*	0.95	0.94	0.93	0.94	0.94	0.94	0.94	0.94	0.94	0.95	0.95
	*hete*	0.95	0.94	0.94	0.93	0.94	0.94	0.94	0.94	0.94	0.94	0.94

We also evaluated the mean, median and standard deviation of the width of the 90% and 95% confidence intervals. The mean, median and standard deviation of the width of the confidence intervals based on the heteroskedastic IV estimator are always smaller than the same measures of the confidence intervals based on the homoscedastic IV estimator. In summary, this simulation study shows that we can use the asymptotic distribution of both estimators based on instruments to construct confidence intervals for the contagion parameter, and that the confidence intervals based on the heteroskedastic IV estimators should be preferred.

## Conclusion

The canonical model of contagion was extended to cases where the errors are heteroskedastic. This is a common case when a country’s stock market index is used as the indicator of the performance of the economy. We showed that in this case, the volatilities are valid instruments and the Cragg-Donald statistics showed they could reduce the bias considerably. Further simulation showed that the bias of the heteroskedastic estimator of the contagion parameter, based on the proposed instruments is zero or almost equal to zero, and that the confidence intervals constructed using the asymptotic distribution of the estimator has a coverage percentage close to the nominal values. We also showed that although the homoscedastic IV estimator also has zero or almost zero bias, the performance of its punctual and confidence estimators are a little worse than the punctual and confidence estimators given by the heteroskedastic IV estimator.

## Supporting Information

S1 File Program in R to build Fig 1. (R)

S2 File Program in R to Simulate Proposition 1. (R)

S3 File Program in R to Generate Results for Tables 3 and 4. (R)
